# Cyanobactins from Cyanobacteria: Current Genetic and Chemical State of Knowledge

**DOI:** 10.3390/md13116910

**Published:** 2015-11-13

**Authors:** Joana Martins, Vitor Vasconcelos

**Affiliations:** 1 Faculty of Sciences, University of Porto, Rua do Campo Alegre, Porto 4169-007, Portugal; E-Mail: joana.o.martins@gmail.com; 2 Interdisciplinary Centre of Marine and Environmental Research (CIIMAR/CIMAR), University of Porto, Rua dos Bragas 289, Porto 4050-123, Portugal

**Keywords:** cyanobacteria, cyanobactins, genetic clusters, chemical structures, bioactivities, ecological roles, biotechnological potential

## Abstract

Cyanobacteria are considered to be one of the most promising sources of new, natural products. Apart from non-ribosomal peptides and polyketides, ribosomally synthesized and post-translationally modified peptides (RiPPs) are one of the leading groups of bioactive compounds produced by cyanobacteria. Among these, cyanobactins have sparked attention due to their interesting bioactivities and for their potential to be prospective candidates in the development of drugs. It is assumed that the primary source of cyanobactins is cyanobacteria, although these compounds have also been isolated from marine animals such as ascidians, sponges and mollusks. The aim of this review is to update the current knowledge of cyanobactins, recognized as being produced by cyanobacteria, and to emphasize their genetic clusters and chemical structures as well as their bioactivities, ecological roles and biotechnological potential.

## 1. Introduction

Cyanobactins belong to the class of ribosomally synthetized peptides with post-translational modifications (RiPPs) that can be generally defined as cyclic peptides containing modifications, which include azole/azoline rings, d-sterocenters and in some cases, prenyl groups [1].

These compounds can be produced by distinct cyanobacteria strains through a pathway recently assigned as post-ribosomal peptide synthesis (PRPS) [2]. In PRPS, an unmodified precursor peptide produced by translation directly encodes the sequence that will form the mature cyanobactin [2,3]. Subsequent cleavage of the precursor peptide is followed by several modifications leading to the formation of the end product [2,4]. The denomination, cyanobactins, was introduced for the first time in 2008, based on the related features and biosynthetic pathways of these cyanobacterial compounds [5]. The first cyanobactins to be described were ulicyclamide and ulithiacyclamide, in the tunicate *Lissoclinum patella* by Ireland and Scheuer in 1980 [6]. Twenty-five years later, two separate studies demonstrated that the cyanobacterium, *Prochloron*, symbiont of the tunicate, was in fact responsible for the production of cyanobactins through a PRPS pathway [7,8]. The cyanobactins are found in different marine animals, such as ascidians, sponges and mollusks. At present, however, it is believed that cyanobactins are derived exclusively from cyanobacteria [9,10]. Interestingly, not all of the currently known cyanobactins can be associated with a cyanobacterial symbiont. This review aims to present the current state of knowledge of cyanobactins, which are produced solely from cyanobacteria, with special emphasis given to their chemical structures, genetic biosynthetic pathways, bioactivities, ecological roles and biotechnological potential.

## 2. Producing Cyanobacterial Strains

It is estimated that 10% to 30% of all cyanobacteria can produce cyanobactins [9,11,12]. These peptides seem to be widespread among symbiotic, as well as free-living cyanobacteria, from terrestrial to freshwater and marine environments [12,13,14]. A recent study analyzed the genome of 126 cyanobacteria strains, which revealed that 31 cyanobactin gene clusters were present in 24% of the strains. Although the cyanobactin genetic clusters appear to be sporadically distributed among cyanobacteria, they are more frequent in *Arthrospira*, *Oscillatoria* and *Microcystis* genera. In contrast, the *Prochlorococcus* or *Synechococcus* genomes seem to lack these cyanobactin gene clusters [11].

**Figure 1:**
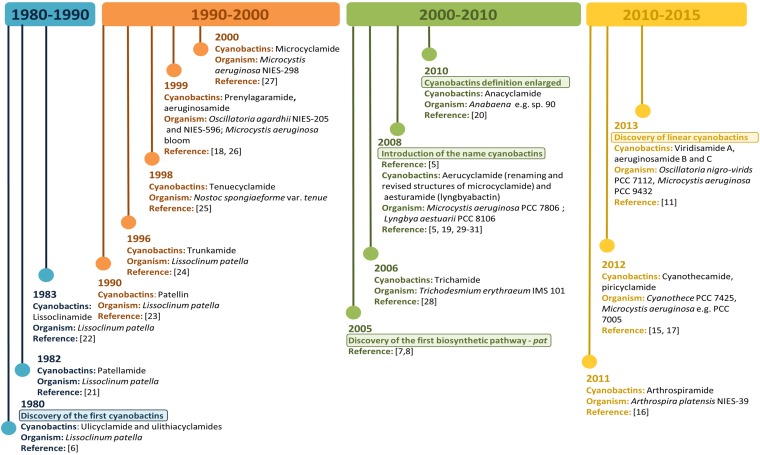
Cyanobactins Time Line. Evolution of cyanobactins since their discovery in 1980 to present.

Thus far, cyanobactin biosynthetic gene clusters and their respective associated metabolites have been described in cyanobacteria belonging to the unicellular genera *Prochloron* (patellamide, lissoclinamides, ulithiacyclamides, patellin and trunkamide) [10], *Microcystis* (microcyclamide, piricyclamide and aerucyclamides) [10,15] and *Cyanothece* (cyanothecamides) [16,17]. Filamentous non-heterocystous genera such as *Trichodesmium* (trichamide) [10], *Planktothrix* (prenylagaramide) [16,18], *Lyngbya* (aesturamide) and *Arthrospira* (arthrospiramide) [5,16,19] and filamentous heterocystous genera such as *Anabaena* (anacyclamide) [20] and *Nostoc* (tenuecyclamide) [10] are also described as cyanobactin producers. Figure 1 shows cyanobactins time line evolution since their discovery to present.

Additionally, cyanobactin genes have been found in distinct cyanobacteria orders [12,13,14] and several cyanobactin genetic clusters with unknown products have been described [11,16]. Therefore, it is important to highlight that the genetic and chemical diversity of the cyanobactins still remains to be explored in greater depth.

## 3. Chemical Structures

Cyanobactins are generally defined as ribosomally synthesized, N-C macrocyclic peptides produced by cyanobacteria [5,9]. Prenylation, or heterocyclization of amino acids, present a second feature of cyanobactins that altogether share a number of characteristics [9]. These compounds are 6–20 amino acids in length. l-amino acids are commonly found in these peptide structures, although more rarely d-amino acids may be present [4,32]. The epimerization to the non-proteinogenic D-form is thought to be spontaneous and not due to enzymatic activity [4,32]. Initially it was assumed that cyanobactins were only composed of cyclic peptides; however, in 2013, the structural diversity of cyanobactins was expanded to encompass highly modified linear peptides with rare post-translational modifications [11].

Cyanobacteria are able to produce cyanobactins that contain heterocyclized amino acids (see Section 5) as well as non-heterocyclized amino acids, which may be sporadically prenylated or geranylated (see Section 6). Cyanobactins that contain heterocycles (see Section 5) present 6–11 amino acids in length and both thiazole (described in 36 cyanobactins) and oxazoline (described in 27 cyanobactins) are frequent in their structures. Thiazoline (described in 16 cyanobactins) and oxazole (described in six cyanobactins) are less frequent. Disulfide bridges are only present in the ulithiacyclamide group—where two of the four encoded cysteines form a disulfide bridge and the other two are heterocyclized to thiazole. In addition, the formation of the disulfide bridge is considered to be spontaneous, owing to the fact that the cyanobactin genetic clusters do not encode an enzyme to carry out this reaction [4,9]. Some cyanobactins may present prenylation of threonine, serine and tyrosine amino acids and, more rarely, *N*-methylation, as is the case for microcyclamide. The linear cyanobactins, such as aeruginosamides and viridisamide A, range in length from 3 to 5 amino acids and present prenylated *N*-termini and methylated *C*-termini bound to thiazoles.

Cyanobactins lacking heterocyclized amino acids (see Section 6) range in length from 7 to 20 amino acids and have a conserved proline residue. The prenyl attachments are present in all cyanobactin families, whereas geranylation may occur in anacyclamide and piricyclamide families. The latter may contain disulfide bridges.

The enormous cyanobactin diversity results from the distinctive genetic features of this class of compounds. The presence of one or more precursor peptide genes with a hypervariable core region certainly contributes to the chemical diversity within this class of compounds [2,9,10].

## 4. Biosynthetic Genetic Clusters

**Figure 2:**
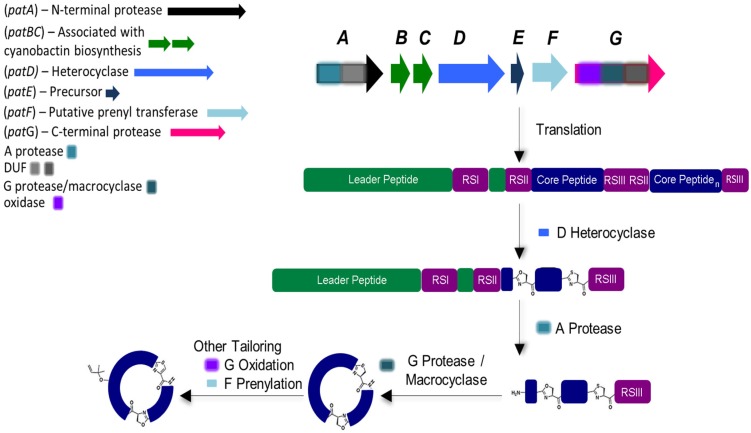
General biosynthesis of cyanobactins that contain azoline heterocycles. Heterocyclization and proteolytic tailoring to produce macrocycles succeed translation of the core peptide. Multiple copies of the core peptide and recognition sequences may exist. Additional tailoring such as prenylation and oxidation/dehydrogenation to azoles may occur. Additional modifications are occasionally present. Adapted with permission from [2] (http://dx.doi.org/10.1039/c2np20085f). Copyright © The Royal Society of Chemistry, 2015.

Cyanobactin genetic clusters (approximately from 8 to 19 kb in length) have only been identified in cyanobacteria [11]. The active cyanobactin gene clusters always encode: (1) two protease genes, *A* (*N*-terminal protease) and *G* (*C*-terminal protease), that are related to *patA* and *patG* genes from the patellamide biosynthetic pathway, also known as *pat* (both *A* and *G* genes comprehend a domain of unknown function (DUF) and a macrocyclase domain which is only present in the G-protease [33]);(2) a precursor peptide gene *E*, homolog to *patE*, which directly encodes the cyanobactin structure that acts as a substrate for post-translational modifications and, (3) two genes *B* and *C*, which are related to *patB* and *patC*, that encode short conserved proteins of an unidentified function [9,10]. Although *B* and *C* genes are conserved among nearly all cyanobactin genetic clusters, studies have demonstrated that these genes are non-essential [34]. In addition, the cyanobactin gene clusters may also encode homologs of PatD and/or PatF, denoted as D-protein (cyclodehydratase) and F-protein (prenyltransferase), as well as thiazoline/oxazoline dehydrogenases (responsible for the aromatization of the heterocycles to thiazoles and oxazoles), methyltransferases and other non-characterized proteins (Figure 2) [10]. The *D* gene is only present in cyanobactin biosynthetic pathways that produce compounds with heterocyclized amino acids (see Section 5). In contrast, the *F* gene seems to be present in all cyanobactin pathways, with the exception of the non-prenylated trichamide (see Section 5 and Section 6). Nevertheless, this gene was proven to be essential for the synthesis of the non-prenylated patellamides, suggesting that it may have a distinct function in this biosynthetic pathway [34].

Cyanobactin biosynthesis begins with the precursor E-peptide, which is composebd of an *N*-terminal conserved leader sequence that is recognized by some of the modifying and cleaving enzymes [35]. However, reports indicate that cyanobactin genetic clusters may also employ more than one precursor peptide. In fact, it has been described that a genetic cluster may contain up to 10 precursor peptide genes [11,16]. Within the E-peptide that contains the enzymes recognition sequences (RSI, RSII and RSIII), 1–4 hypervariable core sequences may be present and dictate the amino acid backbone of the cyanobactin [2,3,9,10,36]. In the presence of more than one core peptide, each one is flanked by *N*-terminal (RSII) and *C*-terminal (RSIII) recognition sequences which may constitute four or five amino acids. In the presence of D-protein, cyclodehydratase, (and in order to form oxazoline and thiazole) heterocyclization of cysteines, serines and/or threonines will occur, directed by the recognition sequence RSI [37,38]. A-protease cleaves the precursor peptide RSII, leaving a free amine available for macrocyclization. Lastly, the G-protease splits the precursor peptide RSIII and catalyzes C-N macrocyclization. Subsequently, other transformations may occur such as: (1) the prenylation of serines/threonines or tyrosines/tryptophans residues, catalyzed by the PatF class of prenyltransferases (Figure 2) [16,39]; (2) the oxidation of heterocycles to oxazoles and thiazoles when the oxidase domain ispresent within the *G* gene or separate [11,33,40] and more uncommonly; (3) geranylation [9].

Cyanobactins have been classified into different groups based on the correspondence between genotypes and chemotypes [16]. Another classification scheme has also been proposed based solely on cyanobactin structures [41].

### 4.1. Heterocyclization

The heterocyclase (cyclodehydratase) accomplishes heterocyclization of cysteine, serine and threonine residues to thiazolines or oxazolines and eliminates water [2,42]. The cyanobactin heterocyclases (D) were studied in detail in the patellamide and trunkamide pathways [37] and reviewed in the broader context of RiPPs [4,42]. The activity of the heterocyclases (D), from both the above-mentioned pathways, is ATP-dependent and occurs in a defined order of reactions [4,37]. An adenylase mechanism has been proposed for TruD, from the trunkamide pathway, whose crystal structure presents as a three-domain protein [43]. The processivity of the enzyme requires the presence of the leader peptide to be attached to the core, indicating that heterocyclization occurs before cleavage and macrocyclization of the precursor peptide [37,42]. A short sequence element in the leader peptide is an indication for heterocycle formation [2,36]. Cyanobactin pathways encoding a heterocyclase may also codify an oxidase domain responsible for oxidation of thiazolines and oxazolines to thiazoles and oxazoles [9,11].

### 4.2. Cleavage and Macrocyclization

Two subtilisin-like proteins, *N*-terminal protease (A) and *C*-terminal protease (G), are encoded in cyanobactin genetic clusters. It was demonstrated that PatA protease (from the patellamide gene cluster) catalyzes the cleavage of the *N*-terminal protease site of the precursor peptide, removing the leader sequence, whereas PatG protease catalyzes the formation of the macrocyclic peptide while removing the *C*-terminal protease signature [8,44]. PatG protease from *Prochloron* was shown to macrocyclize a broad range of synthetic substrates with non-proteinogenic and d-amino acids at a lower rate [38].

The structure of the protease-macrocyclase domains of PatG and PagG (the latter from the prenylagaramide genetic cluster) was determined and the catalytic triad was presented [33,40]. The crystal structure of the macrocyclase domain of PatG shows that a subtilisin fold is present (like in PatA); however, it contains two helices designated by the “macrocyclization” insert, without conservation in terms of length and sequence. The macrocyclase domain is insensitive to the identity of the residues within the core peptide, since PatG acts on RSIII residues and catalyzes C-N macrocyclization [42]. During macrocyclization, the PatA protease removes the amino terminal linked to the core, yielding a free amino terminal, and PatG protease removes the carboxy terminal sequence flanking the core. The cleavage site is protected by the PatG protease preventing access to water and hydrolysis until the transamidation reaction is complete [33,40].

### 4.3. Prenylation, Oxidation and DUF

The presence of the gene encoding the prenyltransferase has been described in both prenylated and non-prenylated pathways [11]. For instance, the prenyltransferase gene is present in the patellamide (*pat*) pathway that generates the non-prenylated patellamides A and C [8]. In contrast, e.g., prenylagaramide (*pag*), trunkamide (*tru*), and aesturamide (*lyn*) pathways, which also encode the prenyltransferase gene, synthesize the prenylated compounds [5,16]. Prenylagaramide contains *O*-prenylated tyrosine [16], whereas trunkamide contains *O*-prenylated threonine and serine [34]. The prenyltransferases from *lyn* (LynF) and *tru* (TruF) pathways have been characterized biochemically [39,45]. LynF prenylates the oxygen atom of a tyrosine residue using dimethylallyl pyrophosphate (DMAPP). The reverse *O*-prenylated tyrosine undergoes spontaneous Claisen rearrangement yielding the ortho-substituted phenol [45]. In contrast, TruF prenylates serine and threonine residues on the hydroxyl side chain [39,45]. The structure of PatF from the patellamide pathway has been determined, revealing that it embraces, as the other prenyltransferases, the classic TIM barrel fold [46]. However, no enzymatic activity was detected, thus remaining consistent with the absence of prenylation in patellamides A and C. A careful examination revealed that it is in the positions usually occupied by conserved and catalytic active Asp and Lys residues that the residues His125 and Met136 are located, thereby justifying the inactivity of PatF [42,46]. It has already been demonstrated that PatF is essential for patellamide production *in vivo* [34] and consequently it must be responsible for another function in this pathway. Further studies will be required to understand the essential role of this protein in the production of the patellamides [46].

The oxidase domain is conserved among PatG homologs and studies reveal that it is FMN dependent [42]. The related thiazoline oxidase, in the microcin pathway, was the subject of a biochemical study [47] and although this enzyme is related in sequence to the patellamide enzymes, the basis of the substrate recognition remains unclear, as microcins are linear and patellamides are macrocycles [42]. Notwithstanding, it has been demonstrated that one homolog of the oxidase domain of PatG is able to oxidize both linear and macrocycle thiazoline-containing compounds [48] while another homolog only has the ability to perform oxidation on a macrocyclic substrate [4,6].

Both PatA and PatG proteases contain a domain of unknown function (DUF) sharing 56% sequence similarity [8,47]. The DUF domains are found in PatA and PatG from the patellamide-like biosynthetic clusters [49]. It is assumed that epimerization follows heterocyclization and precedes oxidation. Although epimerization has been proposed as a possible role for the DUF domain, it has been suggested that this phenomenon is chemically spontaneous, assigning no roles to DUF domains in the patellamide biosynthesis [32,42]. The crystal structure of PatG-DUF has been characterized as a novel fold dimer with two zinc ions. The practical importance of the dimer remains unclear since the residues involved in Zn^2+^ binding (necessary for dimerization) are not conserved among DUF domains of the other homologs; it is subsequently not clear if the other domains are dimers [49]. The DUF domain does not bind to linear substrates (simple peptides or peptides with heterocycles) [47] and additional experiments will be necessary to determine if the DUF domain binds to the macrocycle, or the core peptide alone [42].

## 5. Cyanobactins Encoding Heterocyclization Enzymes

### 5.1. Ulicyclamide, Ulithiacyclamides, Patellamides and Lissoclinamides

At the beginning of the 1980s, interest in compounds produced by didemnid tunicates started increased as a part of some programs to isolate antineoplastic natural products. These marine invertebrates were proven to be good candidates since they harbor unicellular prokaryotic algae. Although nitrogen fixation was not demonstrated in these symbionts at the time, this possibility prompted research to look for novel nitrogenous metabolites in tropical didemnid species [21]. Additionally, cytotoxic compounds were already documented from tunicate extracts [21]. A good example is didemnin B, the first marine natural product evaluated in anticancer clinical trials [50].

**Figure 3:**
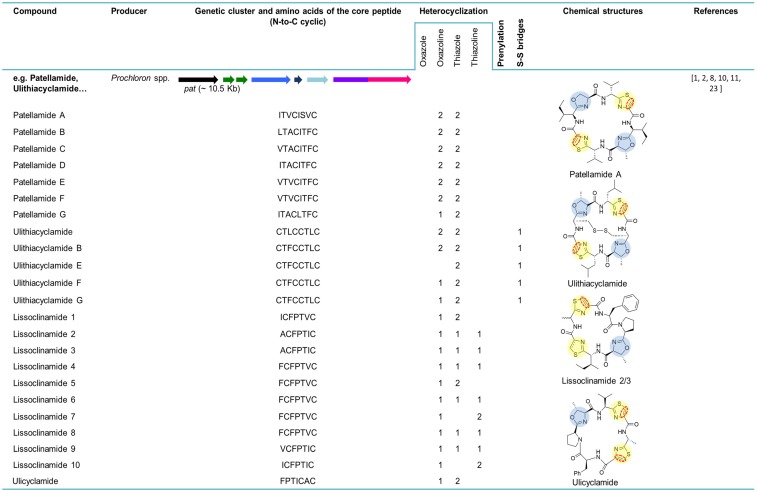

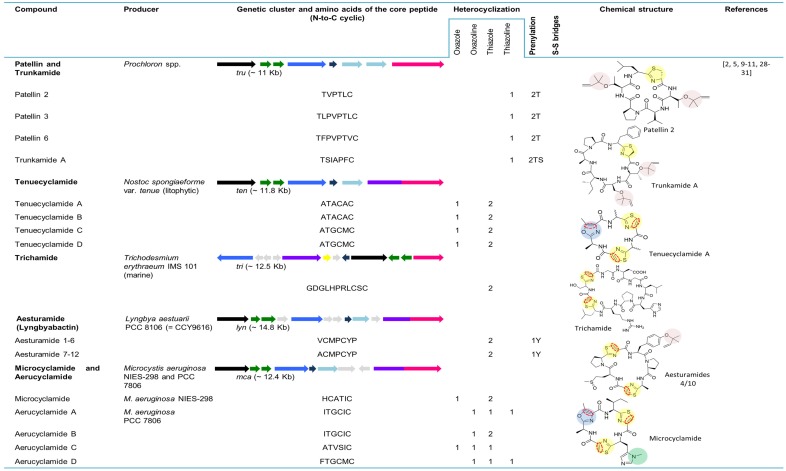

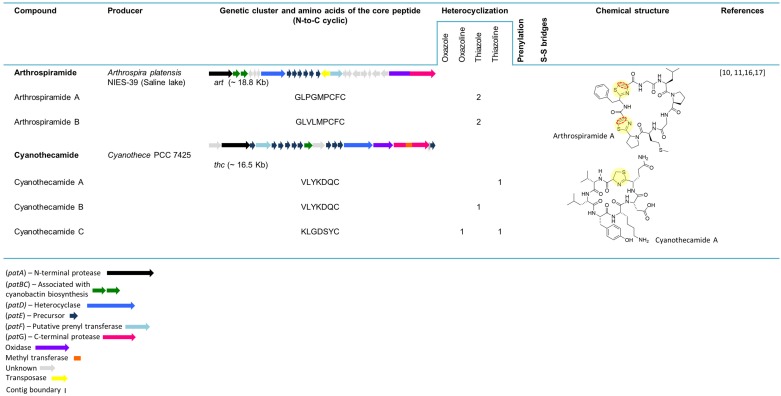
Cyanobactins that encode heterocyclization enzymes. The peptides are organized in chronological order and the sequence of the core peptide is presented in the linear form. The location of the prenyl-group is indicated by one letter amino acid abbreviation in the correspondent column. The genes are identified by different colors. Heterocyclization of Cys (pale yellow) or Ser/Thr (pale blue), oxidation to azole (red dashed line), prenylation (pale red) and *N*-methylation (pale green) are indicated in the chemical structures.

Ulicyclamide and ulithiacyclamide were the first cyanobactins to be discovered by Ireland and Scheuer in 1980 (Figure 3) [6]. These small cyclic peptides isolated from the ascidian *L. patella* from Palau, Western Caroline Islands, unveiled a combination of chemical features, including N-to-C macrocyclization and heterocyclization to form thiazol(in)e and oxazoline motifs that were unique [2,6]. It was unclear at the time if the ascidian hosts or their partners produced these compounds, since information about chemical switches between the ascidians and their symbiotic algae was scarce [6]. Two years later, the octapeptides patellamides A, B and C were reported and isolated from *L. patella* collected at Eil Malk Island, Palau Islands [21]. In 1983, three new cyclic heptapeptides (1, 2 and 3) recently named lissoclinamides from *L. patella* were described as the first thiazoline-containing peptides isolated from this organism [22]. This work developed by Wasylyk and co-workers also presented a revised structure for ulicyclamide. In fact, in 1983, Hamamoto reported the structure of patellamide A (ascidiacyclamide) with a different skeleton [51] and later studies assigned the correct structures of ulicyclamide and patellamide A, as described by Wasylyk and Hamamoto [1,52,53]. In 1989, four novel cyclic peptides, including patellamide D and lissoclinamides 4, 5 and 6 were isolated from the same tunicate species [54,55]. The known peptides derived from *L. patella* were categorized in two separate groups, one constituted by ulithiacyclamide (structure determined by Biskupiak and Ireland [56]) and patellamides A, B and C and the other represented by ulicyclamide and lissoclinamides 1–3. Both groups present uncommon amino acids containing thiazole moieties, but differ in the nature of their macrocyclic ring skeleton [55]. At the same time, Williams and Moore [57] reported on the isolation and structure determination of ulithiacyclamide B from *L. patella* from Pohnpei, Federated States of Micronesia. The difference between ulithiacyclamide and ulithiacyclamide B is that the latter lacks the symmetry associated with ulithiacyclamide and possesses a phenyl group [57]. Patellamide E was isolated from *L. patella* collected in Pulau Salu, Singapore. The tunicate extract also contained patellamides A and B and ulithiacyclamide [58]. Patellamide F was characterized from the extract of *L. patella* sampled from northwestern Australia. The extract also contained patellamide B, ulithiacyclamide and lissoclinamide 3 [59]. In 1998, during a screening for multidrug resistance (MDR) reversing agents from marine organisms, four new cyclic peptides, assigned patellamide G and ulithiacyclamides E−G, were isolated from the tunicate *L. patella* collected in Pohnpei, along with the known patellamides A−C and ulithiacyclamide B. A common aspect of the novel metabolites was that at least one of the threonine units was not cyclized to an oxazoline ring [60].

In 2005, the first cyanobactin gene cluster (assigned as *pat*, originated the patellamide group) (Figure 3) was described in *Prochloron didemni*, the cyanobacterial symbiont of *L. patella* [8]. This discovery allowed the attribution of patellamides to the cyanobacterium *Prochloron* and confirmed that these compounds are synthetized by a ribosomal pathway. The function of the genes was also confirmed by heterologous expression in *Escherichia coli* [8]. At the same time, heterologous production of patellamides D and A (ascidiacyclamide) was verified by shotgun-cloning techniques by cloning *Prochloron* gDNA into an *E. coli* host, affirming that the *Prochloron* sp. is the primary biosynthetic source of the patellamides isolated from *L. patella* [7]. The presence of patellamides A and C was previously reported in the *L. patella* reef sample, but failed to detect any other patellamide variants as a major product of the crude extract. The *pat* gene cluster (~10.5 kb) contains a single precursor peptide gene, *patE*, that encodes for patellamides A (ITVCISVC) and C (VTACITFC). *PatA* and *patG* genes border this genetic cluster while *patD* and *patF* genes surround *patE*. *PatB* and *patC* genes, with an unknown function, are also present in this biosynthetic pathway. At that time, the function of *patF* was not known. Since the *pat* cluster was only found in a patellamide-producing *L. patella* strain, it was suggested that *pat* was probably acquired through horizontal gene transfer [8]. In 2006, 46 *Prochloron*-harboring ascidians were analyzed for the presence of *pat* genes. It was demonstrated that each symbiotic strain contains a unique pathway and that an extended library of patellamides is produced due to small hypervariable cassettes present in a conserved genetic background. Six *patE* gene variants (*E1*–*E6*) encoding seven different cyclic products, including patellamides A, B and C, ulithiacyclamide, ulicyclamide and lissoclinamides 2/3 and 4/5, were found among the analyzed samples. For instance, patellamide C is encoded by *E1* (position I) and *E2* (position II). Additionally, 23 other *patE* variants were found in 96-well plate *E* gene clone libraries [34].

### 5.2. Patellins and Trunkamide

Patellin 2 (Figure 3), a modified cyclic peptide, was characterized from the Fijian marine tunicate *L. patella*. Contrary to the previously identified *Lissoclinum* peptides, this cyanobactin lacks the distinguishing thiazole and oxazoline amino acids and contains a thiazoline and two threonine residues modified as dimethylallyl ethers [23]. The cyclic compounds patellins 1–6 and trunkamide A were isolated in 1996 from *Lissoclinum* spp. collected in Viti Levu, Fiji (patellins 1–5) and in the Great Barrier Reef, Australia (patellins 3, 5 and 6 (G) and trunkamide). While patellins are hexa (patellins 1–2) or octa (patellins 3–5 and 6 (G)) peptides, trunkamide is a heptapeptide [24]. The presence of two dimethylallyl threonines (or one threonine and one serine) side chains and one thiazoline ring in the backbone of the patellins is the most important feature of these compounds [24].

Trunkamide A (Figure 3) was synthesized and its stereochemistry was revised in 2000 by Wipf and Uto [61]. The interesting activities of trunkamide A (see Section 7) further led to large-scale production of trunkamide A and enabled the completion of its biological activity as well as conformational studies. To that end, a total solid-phase synthesis and preliminary characterization of trunkamide A was performed and the configuration proposed by Wipf was confirmed [62]. In 2001, a solution was published by Mckeever and Pattenden [63] for the synthesis of trunkamide A. In order to obtain structure-activity relationships, the three-dimensional structure (solution structure) of this peptide was presented [64].

The patellin pathway *tru* (~11 kb) (Figure 3) directly encodes on the *tru* gene patellins 2 and 6 and was discovered in 2008 by Donia and co-workers [5]. The *tru* genetic cluster was cloned in *E. coli*, confirming that the same one was responsible for the biosynthesis of the prenylated compounds. As was seen in the *pat* gene cluster, hypervariability in small cassettes within the *E* gene led to the diversity of natural cyclic peptides. Variants of the *E* gene from the *tru* pathway were cloned from the ascidian samples containing patellins and trunkamide, revealing two new genes *truE2* and *truE3*. While TruE2 encodes patellin 6 and trunkamide, TruE3 encodes three copies of trunkamide. The *tru* genetic cluster is syntenic to *pat*; however, two major differences are perceived: (1) *truG* does not contain an oxidase domain, which is congruent with the fact that patellins have thiazolines, whereas patellamides are oxidized to thiazoles; and, (2) two copies of *truF* located in the *tru* gene cluster are contiguous to each other. It was suggested that *F* or *D* genes were responsible for the heterocyclization of serine and threonine in the patellamides and/or prenylation in patellins, since the leading differences between *pat* and *tru* genetic clusters were detected in these genes [5].

### 5.3. Tenuecyclamides

In 1998, Banker and Carmeli announced the discovery of tenuecyclamides (A−D). These four hexapeptides, harboring two thiazoles and one oxazole, were isolated from the litophytic cyanobacterium strain *Nostoc spongiaeforme* var. *tenue* (TAU strain IL-184-6) [25]. The cyanobactin genetic cluster for tenuecyclamides, *ten* (~11.8 kb) was described from the same strain. The number and organization of the genes in this biosynthetic pathway is equal to that in the *pat* pathway. A unique feature of this gene cluster is its precursor peptide (TenE) that encodes four copies of the amino acid sequences of the core peptide, two for each of the hexapeptides A and C (Figure 3) [5]. Tenuecyclamide B differs from A only in stereochemistry [25].

### 5.4. Trichamide

Trichamide was the first natural product reported from the marine bloom-forming cyanobacterium *Trichodesmium erythraeum* ISM101 in 2006. The previous discovery of the patellamide gene cluster [8] contributed to the identification of a related cluster in this cyanobacterium strain and enabled the prediction of the *tri* biosynthetic pathway through the whole genome sequencing. This peptide, cyclized by an N-C terminal amide bond, comprises 11 amino acids, including two cysteine-derived thiazole groups (Figure 3). Contrary to *pat*, the *tri* gene cluster has a bidirectional gene order and the oxidase domain encodes separately from the protease gene. This gene cluster (~12.5 kb) consists of a unique precursor peptide gene encoding a single copy of the trichamide core peptide, a cyclodehydratase, an oxidase, two proteases and hypothetical genes. The presence of tRNA synthetase genes bordering the *tri* gene cluster proposed the possible procurement of this gene cluster through the horizontal gene transfer [28].

### 5.5. Aesturamides (Lyngbyabactins)

The genome mining of *Lyngbya aestuarii* CCY9616 (PCC 8106) by Donia and colleagues [5] allowed for the identification of a new cyanobactin genetic cluster dubbed *lyn* (~14.8 kb). This contains all of the homologs genes present in the patellamide genetic cluster (*A*–*G*), with the exception of the occurrence of four domains of unknown function. The possible new cyclic peptides structures were predicted based on the single *lynE* sequence that contains one copy of the Ala-containing sequence and two copies of the Val-containing sequence [5]. Twelve cyclic peptides assigned to aesturamides were described as a result of the single precursor peptide present in the *lyn* genetic cluster, representing the highest number of natural products produced by a ribosomal pathway. Aesturamides are synthetized enzymatically as reverse *O*-prenylated tyrosine ethers that subsequently undergo a Claisen rearrangement to produce forward *C*-prenylated tyrosine [19]. Two different groups of aesturamides arise from the two different sequences of the core peptide. Aesturamides 1 to 3 are the result of post-translational modifications of the *lynE* sequence, VCMPCYP (Figure 3). These compounds are reverse *O*-prenylated, non-prenylated and forward *C*-prenylated. Aesturamides 4 to 6 belong to this first group and are sulfoxide derivatives of 1–3. Aesturamides 7 to 12, the products of the *lynE* sequence ACMPCYP, are in the same manner as the above-mentioned aesturamides, reverse *O*-prenylated, non-prenylated and forward *C*-prenylated compounds, with or without Met oxidation. Aesturamides are N-C cyclic compounds, containing thiazole, which is congruent with the presence of *lynA*, *lynG* and *lynD* genes and an oxidase domain [19].

### 5.6. Microcyclamides and Aerucyclamides

Microcyclamide was the first cyclic hexapeptide composed of five-membered heterocycles (Figure 3) reported from the bloom-forming cyanobacterium strain *Microcystis aeruginosa* NIES-298, by Ishida and co-workers in 2000 [27]. Later, it was demonstrated that microcyclamide is a product of a ribosomal pathway, through the activity of a set of enzymes that are related to the ones involved in patellamide biosynthesis [31]. Genomic data analysis of *M. aeruginosa* PCC 7806 allowed for the identification of a gene cluster encoding proteins with more that 90% similarity to the nine proteins encoded in NIES-298 (~12.4 kb) and resulted in the discovery and isolation of two new microcyclamide family peptides: microcyclamide 7806A and microcyclamide 7806B. The genes from the cyanobactin genetic clusters (*mca*) in both strains have the same order as the ones from the patellamide gene cluster, with the exception of two open-reading frames with no assigned functions. The *E* gene in NIES-298 contains two-peptide coding regions (HCATIC; (Figure 3)), which is consistent with the amino acid composition and order in the microcyclamide molecule, whereas the corresponding gene in PCC 7806 encodes four (ITGCIC, ITGCIC, ATVSIC and FTGCMC; (Figure 3)). Microcyclamide 7806A and 7806B (later renamed aerucyclamide C and D) structures are assigned to the precursor sequences ATVSIC and FTGCMC (Figure 3) [31]. Subsequently, microcyclamide structures were revised and renamed. Aerucyclamides A−D were isolated from *M. aeruginosa* PCC 7806 [29,30]. It was proposed that they were the actual ribosomal-encoded metabolites, which is consistent with the genetic data provided by Ziemert [31]. More recently, microcyclamide-like genetic clusters were also detected in different *Microcystis* strains [11].

### 5.7. Arthrospiramide

The *art* gene cluster (~18.8 kb) (Figure 3) was uncovered from the genome of the economically important cyanobacterium *Arthrospira platensis* NIES-39 [16]. The organization of *art* (from *A* to *G* gene) is similar to that of the most common cyanobactin genetic clusters, with two exceptions: (1) it contains six precursor peptides that encode different products and (2) a transposase is present between *artE* and *artF* genes. Closely related *art* gene clusters were also found in the genome of *A. platensis* strain Paraca and an edible *Arthrospira* strain. In the first case, all the cyanobactin genes are present with the exception of the *E* genes, since the genome was highly fragmented on small contigs. The genome of the second *Arthrospira* strain contains three additional precursor peptides encoding for different products. In an attempt to isolate peptides from this genera, commercial preparations of dried *A. platensis* were acquired, and the compounds derived from the ArtE3 and ArtE5 precursor peptides were partially purified and named arthrospiramide A and B, respectively [16]. Recently, several arthrospiramide-like genetic clusters from different *Arthrospira* strains were uncovered through genome-mining techniques [11].

### 5.8. Cyanothecamides

Three new cyanobactins, (cyanothecamides (A−C)), were discovered from a culture of *Cyanothece* sp. PCC 7425 exposed to a heat shock [17]. The cyanothecamide gene cluster, *thc* (~16.5 kb) (Figure 3), was previously reported in [16], but no cyanobactins were detected in cultures growing in normal conditions over a two-year period. The organization of this genetic cluster is unique (representing a separate cyanobactin genotype), including a methyltransferase and an ABC transporter. Furthermore, nine copies of the *thcE* gene are present, each one encoding a different product. Among three plasmids present in the *Cyanothece* sp. PCC 7425, the tenth *thcE* gene was detected in the plasmid pP742501 [16]. The *thcE2* gene, from the cyanothecamide gene cluster, was identified as the ortholog of *patE*. The ThcE2 cassette, VLYKDQC, is responsible for the synthesis of cyanothecamides A and B. Cyanothecamide C, detected by mass spectrometry (MS), was assigned to *thcE3* gene with the coding sequence KLGDSYC and revealed a new cassette-flanking sequence (SNCIG), which has not yet been described in the canonical *patA* recognition sequence. Cyanothecamides are macrocycle heptapeptides with a single sulfur heterocycle that gives this group of compounds a novel backbone arrangement [17].

### 5.9. Aeruginosamides and Viridisamide—Linear Cyanobactins

**Figure 4:**
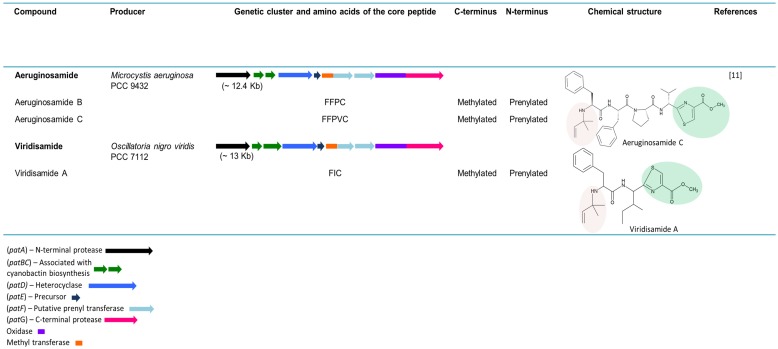
Linear cyanobactins. The genes are identified by different colors. Prenylated *N*-termini (pale red) and methylated *C*-termini bound to thiazoles are highlighted in the chemical structures.

In 2013, the genome mining of 126 cyanobacterial strains, led to the discovery of linear cyanobactins by Leikoski and colleagues [11]. The cyanobactin genetic clusters of *M. aeruginosa* PCC 9432 (~12.4 kb), *Oscillatoria nigro-viridis* PCC 7112 (~13 kb) and *Leptolyngbya* sp. PCC 7376 (~11.3 kb) (Figure 4), encode an unusual bimodular protein with homology to SAM-dependent methyltransferases and prenyltransferases, thereby indicating that a new cyanobactin variety could arise as an end product. However, in *Leptolyngbya* sp. PCC 7376, the cyanobactins could not be predicted since the identified precursors lacked the recognizable cleavage sites. The bioinformatics study predicted the presence of the linear cyanobactins in *M. aeruginosa* PCC 9432 and *O. nigro-viridis* PCC 7112 due to the presence of conserved cleavage sites. The tetrapeptide aeruginosamide B and the pentapeptide aeruginosamide C were detected in *M. aeruginosa* PCC 9432. A similar cytotoxic compound, named aeruginosamide, was previously reported from a *M. aeruginosa* bloom [26]. Both aeruginosamide B and C present a prenylated *N*-termini and a methylated *C*-termini bound to thiazole. In *O. nigro-viridis* PCC 7112, the linear tripeptide viridisamide A was identified. As in aeruginosamides B and C, viridisamide A contains a prenylated *N*-termini and a *C*-termini bound to thiazole [11].

## 6. Cyanobactins Non-Encoding Heterocyclization or Oxidation Enzymes

### 6.1. Anacyclamides

**Figure 5:**
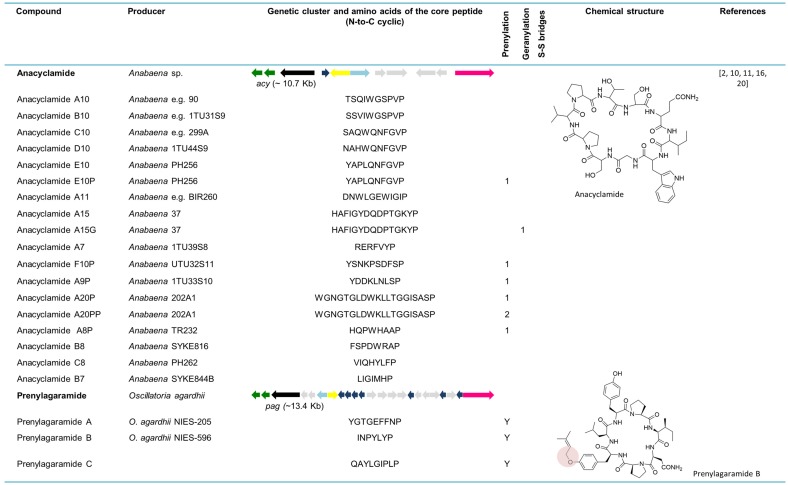

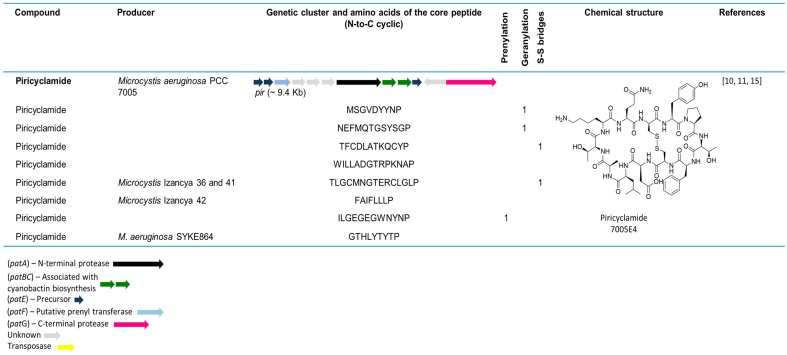
Cyanobactins that do not encode heterocyclization or oxidationenzymes. Thepeptides are organized in chronological order and the sequence of the core peptide is presented in linear form. The location of the prenyl group is indicated by one-letter amino acid abbreviation in the corresponding column. The genes are identified by different colors. Prenylation (pale red) is indicated in the chemical structure.

The whole genome sequence from the strain *Anabaena* sp. 90 allowed for the discovery of new cyanobactins, designated anacyclamides by Leikoski and colleagues [20]. Using bioinformatics tools coupled with stable isotope labeling and MS, these novel cyclic peptides were found in a total of 27 *Anabaena* strains. Anacyclamides can differ from 7 to 20 amino acids in length, enclosing only proteinogenic amino acids that can be prenylated or geranylated. The *acy* gene cluster (~10.7 kb) is composed of 11 genes organized bidirectionally, such as in the trichamide gene cluster. Only one precursor peptide gene is present in the *acy* genetic cluster. Nevertheless, 15 distinct amino acid sequences from the core peptide were detected from 27 strains yielding 18 unique anacyclamides. The *acy* gene cluster lacks *acyD* as well as an oxidase domain, which is consistent with the chemical structure of these metabolites. In contrast to the other reported cyanobactins, anacyclamides exhibit a pronounced amino acid variation with only one proline at the conserved *C*-terminus [20]. With the discovery of anacyclamides, the definition of cyanobactins was extended to include unmodified peptides that can have prenylated or geranylated amino acids (Figure 5).

### 6.2. Prenylagaramides

The isolation and structural explanation of the Tyr *O*-prenylated cyclic peptides prenylagaramides A and B were reported in 1999 [18]. The nonapeptide, prenylagaramide A was isolated from *Oscillatoria agardhii* NIES-205, whereas the heptapeptide prenylagaramide B was obtained from a culture of *O. agardhii* NIES-596. The prenylagaramide genetic cluster *pag* (~13.4 kb) in *Planktothrix agardhii* NIES-596 (foremost identified as *O. agardhii* NIES-596) is highly similar to *acy*, as it lacks the heterocyclase domain. Seven precursor peptide genes are present in the *pag* genetic cluster. The E6 gene (INPYLYP) directly encodes prenylagaramide B, while the E7 gene (QAYLGIPLP) encodes the new prenylagaramide C [16]. The difference between prenylagaramides and anacyclamides is that the latter are unadorned N-C cycles, without Tyr *O*-prenylation (Figure 5) [16].

### 6.3. Piricyclamides

The investigation of the inactive cyanobactin gene cluster, *mae*, in *M. aeruginosa* NIES-843 (~19.7 kb) [16] resulted in the discovery of the piricyclamides in *M. aeruginosa* PCC 7005 (Figure 5) [15]. This new cyanobactin family is structurally diverse since some piricyclamides consist exclusively of proteinogenic amino acids, whereas others contain disulfide bridges, such as ulithiacyclamides, and some are prenylated or geranylated. Piricyclamides can differ from 7 to 17 amino acids in length. The *pir* gene cluster in *M. aeruginosa* PCC 7005 (~9.4 kb) is similar to the previously described gene cluster in NIES-843 [16], although the re-examination of the latter has revealed the presence of two extra precursor genes. The inactivation of the gene cluster in NIES-843 is due to a frameshift mutation in *pirG* gene and the presence of two insertion elements in the *pirA* gene. The *pir* genetic clusters constitute one to four precursor peptide genes. Additionally, the analysis of a bloom sample composed of *Aphanizomenon* (97% of the 16S rRNA genes sequenced) and *Microcystis* (3% of the 16S rRNA genes sequenced) revealed 19 different precursor peptide genes disclosing the vast genetic and concomitant chemical diversity of these compounds in a single environmental sample. In this study, 13 different piricyclamide core gene sequences were uncovered in seven *Microcystis* strains, whereas nine piricyclamides were detected by LC-MS in six strains. In strain PCC 7005, all the essential genes for cyanobactin production are present in the *pir* gene cluster and, similar to *acy*, the *D* gene and *G* gene oxidase domains are lacking. Four piricyclamides were found in PCC 7005, two of which are geranylated and one being composed of a disulfide bridge. The PCC 7005 *pir* gene cluster was cloned into *E. coli* from the beginning of *pirE3* and only one piricyclamide TFCDLATKQCYP (*pirE4*) was detected. This was due to the lack of promoters and some precursor genes in the amplified part of the genetic cluster. Phylogenetic inferences using concatenated proteases A and G clearly differentiate piricyclamides from the other *M. aeruginosa* cyanobactins, microcyclamides (Figure 5) [15]. Furthermore, several piricyclamide-like genetic clusters were uncovered for different *Microcystis* strains through genome mining [11].

## 7. Bioactivities

Cyanobactins have various activities derived from their different structures (Table 1). The search for anticancer compounds resulted in the discovery of several cyanobactins, although cytotoxicity has been the most pronounced activity attributed to these peptides (see Table 1) [9,10].

The *in vitro* antitumor activity against L1210 murine leukemia cell cultures was evaluated for the cyanobactins; ulithiacyclamide, ulicyclamide, patellamides A−C and lissoclinamides 1–3 [21,22,51]. The most effective cyanobactin was found to be ulithiacyclamide, with an IC_50_ value of 0.35 µg/mL, followed by patellamides B, C and A, with IC_50_ values of 2.0, 3.2 and 3.9 µg/mL, respectively. Ulicyclamide was the least active with an IC_50_ value of 7.2 µg/mL [21]. Lissoclinamides 1, 2 and 3 exhibited borderline cytotoxicity, with IC_50_ values higher than 10 µg/mL [22,51]. Similarly, patellamide D and lissoclinamides 4–6 displayed slight cytotoxicity against PS lymphocytic leukemia cells with ED_50_ values (µg/mL) of 11, 10, 12 and 6.9, respectively [22]. The intriguing structures and cytotoxic activities reported for cyanobactins led to the discovery of the importance of the oxazoline function that was believed to play an essential role in cytotoxicity. The confirmation of the efficacy of ulithiacyclamide against L1210 murine leukemia cells, with cytotoxic values comparable to the ones from the clinical anticancer drugs, suggested that the potency of this cyanobactin was related to the unique disulfide bridge that supports the conformation of this compound [65]. The mechanistic characteristics involved in the cytotoxicity of ulithiacyclamide were studied by Shioiri and co-workers [66], and it was found that ulithiacyclamide inhibited cell growth in a self-destructive manner by inhibiting RNA and protein synthesis. Additionally, cytotoxicity of this compound against L1210 cells was synergistically enhanced when tested together with bleomycin, an anticancer drug with a different cytotoxic mechanism [66]. The confirmation of ulicyclamide cytotoxicity against L1210 cells (IC_50_ = 13 µg/mL) and its synthetic intermediate lacking an oxazoline function in its structure (IC_50_ = 35 µg/mL), contradicted the earlier idea that oxazoline was essential in cytotoxicity and related the same to its inhibitory effects on both DNA and RNA syntheses [67]. The cyanobactins ulithiacyclamide and ulithiacyclamide B, as well as patellamides A−C, were also tested against the KB cell line. Ulithiacyclamide (IC_50_ = 35 ng/mL) and ulithiacyclamide B (IC_50_ = 17 ng/mL) were found to be the most cytotoxic against the KB cell line, whereas patellamides A (IC_50_ = 3000 ng/mL), B (IC_50_ > 4000 ng/mL) and C (IC_50_ = 6000 ng/mL) were at least two orders of magnitude less active. Nevertheless, all the aforementioned cyanobactins were less toxic than other tested compounds. Ulithiacyclamide B and patellamides A−C were also tested against distinct tumor cell lines such as leukemia (murine L1210), solid tumor (murine colon adenocarcinoma C38 and human colon adenocarcinoma H116) and low malignancy (murine) cell lines. None of the compounds showed selective cytotoxicity against solid tumor cell lines. Ulithiacyclamide B presented similar cytotoxicity against all the tested cell lines, suggesting that this compound is a general cytotoxin and probably too toxic to be active against solid tumors *in vivo*. The patellamides presented very weak activity against the L210 cell line. Ulithiacyclamide B and patellamides B and C did not show selective activity against either L1210 or an acute myelogenous leukemia (C1498) compared with normal myeloid-committed stem cell (CFU-GM). This suggests that none of these compounds would reduce myelo suppression or have a distinct advantage over any clinically used agent if found to be active *in vivo* [57]. In fact, studies on ulithiacyclamide were interrupted due to its extreme cytotoxicity in animal models [68]. The capability of patellamide E as a possible cytotoxic agent was also determined against human colon tumor cells *in vitro*, but the results suggested weak cytotoxicity (IC_50_ = 125 µg/mL) [58]. When tested in the NCI 60 human tumor cell line panel, patellamides B and F and ulithiacyclamide exhibited modest to general cytotoxicity (average LC_50_ values of 48 µM, 13 µM and 3 µM, respectively) without a pattern of differential cytotoxicity of sufficient significance to warrant further studies based on the NCI screen [59].

**Table 1:** Bioactivities of cyanobactins produced from cyanobacteria.

Source Organism	Compound	Bioactivity	References
*Lissoclinum patella* (*Prochloron* spp.)	Lissoclinamides 1–3	Borderline cytotoxicity against L1210 murine leukemia cells (IC_50_ > 10 µg/mL)	[22]
	Lissoclinamides 4–6	Slight cytotoxicity against PS lymphocytic leukemia cells (ID_50_ = 10, 12 and 6.9 µg/mL for lissoclinamides 4, 5 and 6, respectively)	[22]
	Patellamide A	Mild cytotoxicity against L1210 murine leukemia cells (IC_50_ = 3.9 µg/mL)	[21,57]
		Poor cytotoxicity against KB cell line (IC_50_ = 3000 ng/mL)	
	Patellamide B	Mild cytotoxicity against L1210 murine leukemia cells (IC_50_ = 2.0 µg/mL)	[21,57,59,60]
		Poor cytotoxicity against KB cell line (IC_50_ > 4000 ng/mL)	
		General cytotoxicity in NCI’s 60 human tumor cell line panel (Average LC_50_ = 48 µM)	
		Multidrug reversing activity	
	Patellamide C	Mild cytotoxicity against L1210 murine leukemia cells (IC_50_ = 3.2 µg/mL)	[21,57,60]
		Poor cytotoxicity against KB cell line (IC_50_ = 6000 ng/mL)	
		Multidrug reversing activity	
	Patellamide D	Slight cytotoxicity against PS lymphocytic leukemia cells (ID_50_ = 11 µg/mL)	[22]
		Multidrug reversing activity	
	Patellamide E	Weak cytotoxicity against human colon tumor cells (IC_50_ = 125 µg/mL)	[58]
	Patellamide F	General cytotoxicity in NCI’s 60 human tumor cell line panel (Average LC_50_ = 13 µM)	[59]
	Patellin 6	Moderate cytotoxic against P388, A549, HT29 and CVI cells (Average IC_50_ = 2 µg/mL) and inhibition of topoisomerase II activity (IC_50_ = 2.5µg/mL)	[24]
	Trunkamide A	Active against P-388 mouse lymphoma, A-549 human lung carcinoma, HT-29 human colon carcinoma (IC50 = 0.5 µg/mL) and MEL-28 human melanoma (IC50 = 1.0 µg/mL) cell lines.	[69]
	Ulicyclamide	Poor cytotoxicity against L1210 murine leukemia cells (IC_50_ = 7.2 µg/mL)	[21]
	Ulithiacyclamide	Cytotoxicity against L1210 murine leukemia (IC_50_ = 0.35 µg/mL) and KB (IC_50_ = 35 ng/mL) cell lines	[21,57,59]
		General cytotoxicity in NCI’s 60 human tumor cell line panel (Average LC_50_ = 3 µM)	
	Ulithiacyclamide B	Cytotoxicity against KB cell line (IC_50_ = 17 ng/mL)	[57]
*Microcystis aeruginosa*	Aerucyclamides	Toxic to freshwater crustacean *Thamnocephalus platyurus* (LC_50_ = 30.5 µM for aerucyclamide A and LC_50_ = 33.8 µM for aerucyclamide B)	[29,30,31]
		Antimalarial (aerucyclamide B presented IC_50_ = 0.7 µM, aerucyclamide C presented IC_50_ = 2.3 µM and aerucyclamide D presented IC_50_ = 6.3 µM)	
		Aerucyclamide C—moderate activity against *Trypanosoma brucei rhodesiense* (IC_50_ = 9.2 µM)	
		No inhibitory activity against HeLa cells and standard antiproliferative, antibacterial and antifungal assays	
*Microcystis aeruginosa* NIES-298 (freshwater)	Microcyclamide	Slight cytotoxicity against P388 murine leukemia cells (IC_50_ = 1.2 µg/mL)	[27]
*Microcystis* sp.	Microcyclamide	Microcyclamide MZ602—mild cytotoxicity against Molt4 leukemia cell line (20% cell grow inhibition) and mild inhibition of chymotrypsin (IC_50_ = 75 µM)	[70]
		Microcyclamide MZ568—strong cytotoxicity against Molt4 leukemia cell line (36% cell grow inhibition) and no inhibition of serine proteases	
*Nostoc spongiaeforme* var. *tenue* (litophytic)	Tenuecyclamide A, C and D	Inhibited division of sea urchin embryos *Paracentrotus lividus* (ED_100_ = 108 µM, for tenuecyclamide A, ED_100_ = 9.0 µM for C and ED_100_ = 19.1 µM for D). B not tested.	[25]
*Trichodesmium erythraeum* IMS 101 (marine)	Trichamide	No effects found (tested for cytotoxicity, antifungal, antibacterial and antiviral activities)	[28]

Since an MDR phenotype is the problem associated with many chemotherapeutic administrations, cyanobactins have been evaluated for their potential as MDR inhibitors [9]. Patellamide D was the first compound to demonstrate this activity, improving the effectiveness of certain drugs in human cell lines (CEM/VLB 100) as a selective antagonist to MDR [9]. Anti-MDR activity against vinblastine-resistant CCRF-CEM human leukemic lymphoblasts was evaluated for patellamides A−C and G and ulithiacyclamides E−G. Patellamides B and C were considered significantly active in reducing the drug resistance by about ten-fold (the IC_50_ value for vinblastine in the presence of 2.5 µg/mL of patellamides B and C was 12 nM) [60]. Antimalarial activity of several cyanobactins has been also determined. Some patellamides present antimalarial activity even at doses ten-fold greater than the cytotoxic dose [9].

Patellins 1–6 and trunkamide A were also tested for their potential bioactivity in several cytotoxicity assays. While patellins 1–5 and trunkamide were inactive, patellin 6 displayed moderate cytotoxicity against P388, A549, HT29 and CV1 cells (IC_50_ = 2 µg/mL) and inhibition of topoisomerase II activity (IC_50_ of 2.5 µg/mL) [24]. In contrast to the initial findings, trunkamide A appeared to have quite promising antitumor activity [61], exhibiting the antitumor activity against cell lines derived from human tumors. Trunkamide was active against the P-388 mouse lymphoma (IC_50_ = 0.5 µg/mL), A-549 human lung carcinoma (IC_50_ = 0.5 µg/mL), HT-29 human colon carcinoma (IC_50_ = 0.5 µg/mL) and MEL-28 human melanoma (IC_50_ = 1.0 µg/mL) cell lines. Trunkamide A, as well as an acceptable pharmaceutical composition and carrier of this compound, were applied for patent by Bowden and Gravalos [69]. Trunkamide A was initially selected for further testing by the National Cancer Institute (NCI) due to a good COMPARE correlation analysis and specificity against the UO-31 renal cell line (an MDR line). This could suggest a potential novel mechanism of action and a possible non-MDR activity, as well [62,64]. Trunkamide was classified as a pre-clinical candidate by the marine natural products pharmaceutical company, PharmaMar, but no additional information has been accessible in the past twelve years [9].

The effect of tenuecyclamides A, C and D isolated from *N. spongiaeforme* var. *tenue* was determined by the division of the sea urchin embryos *Paracentrotus lividus*. Tenuecyclamide A inhibited the division of sea urchin embryos with ED_100_ of 108 µM, tenuecyclamide C with ED_100_ of 9.0 µM and tenuecyclamide D with ED_100_ of 19.1 µM [25].

Crude methanol extracts from *T. erythraeum*, tested for general cytotoxicity (HTC-116, CEM-TART) and antihuman immunodeficiency virus, antifungal (*Candida albicans*) and antimicrobial (*Staphylococcus* *aureus* and *Enterococcus faecium*) had no significant activity in these assays. The extracts from *T. erythraeum* IMS101 caused neurotoxicity in a mouse assay but the purified trichamide was not the active compound. Trichamide is not released in significant amounts to the cyanobacterial growth medium, suggesting that this cyanobactin may have an antipredation function [28].

Microcyclamide isolated from *M. aeruginosa* NIES-298 exhibited slight cytotoxic activity against P388 murine leukemia cells (IC_50_ = 1.2 µg/mL) [27]. Aerucyclamide 7806A and 7806B (previously designated as microcyclamides) from *M. aeruginosa* PCC 7806 were screened against HeLa cells and standard antiproliferative, antibacterial and antifungal assays, but no inhibitory activity was detected [31]. Aerucyclamides A and B were also tested in a 24-h acute toxicity test assay using the sensitive freshwater crustacean *Thamnocephalus platyurus*. Both peptides were toxic with LC_50_ values of 30.5 and 33.8 µM, respectively. However, it is unclear if this toxicity is ecologically relevant [29]. In search of cyanobacterial compounds against tropical diseases, aerucyclamides A−D were screened against *Plasmodium falciparum* K1, *Trypanosoma brucei rhodesiense* STIB 900, and rat myoblast L6 cells. Aerucyclamide B was the most active compound against the chloroquine-resistant strain K1 of *P. falciparum* (IC_50_ = 0.7 µM), presenting a large selectivity for the parasite in relation to the L6 rat myoblast cell line (IC_50_ = 120 µM). Additionally, an interesting fact was that the antiplasmodial activity decreased by one order of magnitude by one structural modification from aerucyclamide B to A (IC_50_ = 5.0 µM), *i.e.*, reduction of thiazole to a thiazoline residue. Aerucyclamides C and D also presented low micromolar activity (IC_50_ values of 2.3 and 6.3 µM, respectively). All compounds displayed very weak to no toxicity for the L6 cell line (IC_50_ values ranging from 106 to >168 µM). Aerucyclamide C was the most active compound against *T. brucei rhodesiense*, with moderate activity (IC_50_ = 9.2 µM). Aerucyclamides, particularly aerucyclamide B, displayed preferably potent and selective antiplasmodial activity against *P. falciparum* K1 and a large selectivity over the mammalian L6 cell line. Aerucyclamide C exhibited low cytotoxicity against the crustacean grazer *T. platyurus* (LC_50_ = 70.5 µM) [30]. The biological activities of microcyclamides MZ602 and MZ568 were tested against the serine proteases trypsin, thrombin, chymotrypsin and elastase, and against the Molt4 (leukemia) cell line. Microcyclamide MZ602 presented very mild cytotoxicity against the Molt4 cell line (20% cell grow inhibition at 83 µM) and mild inhibition of chymotrypsin (IC_50_ = 75 µM). In contrast, microcyclamide MZ568 displayed a stronger cytotoxicity against the Molt4 cell line (36% cell grow inhibition at 1.8 µM) and no inhibition in the tested serine proteases [70].

Not all the cyanobactins have been evaluated for their bioactivity. Lack of information, especially concerning the cyanobactins discovered in the last several years, is due to the fact that bioactivity assessment is greatly dependent on the available amounts of cyanobactins. Integration of biosynthetic pathways with heterologous expression systems for cyanobactins may lead to the production of sufficient amounts providing new insights for its bioactivity.

## 8. Ecological Roles

Cyanobactins’ ecological functions have not been clearly studied, although they may be related to some of the bioactivities previously described, such as antibacterial activity and allelopathy.

Cyanobactin-like peptides, such as aeruginazoles [71,72] and kawaguchipeptin B [73], isolated from *Microcystis* sp. strain IL-323 and a *Microcystis* bloom showed antibacterial activities. The putative cyanobactins, nostocyclamide and nostocyclamide M isolated from the free-living cyanobacterium *Nostoc* sp. 31 showed allelopathy against competing cyanobacteria and small grazing organisms [74,75]. The allelopathic activity of nostocyclamides suggested that these molecules were essential for competition among cyanobacteria. For this reason, allelopathy emerged as a possible ecological role for cyanobactins. Nevertheless, more studies using other strains and species need to be done before extrapolations are made.

The metal-binding proprieties of some cyanobactins have been pointed out as a possible role for these peptides in nature. This subject has been well documented in several reviews [9,68,76,77,78,79] and therefore we will only shortly summarize some of the most significant work that has been done to date. Most of the cyanobactin metal-binding studies have focused on cyclic hepta- and octapeptides of the lissoclinamide and patellamide groups. Cyanobactins, such as ascidiacyclamide isolated from the ascidian *L. patella* [53], lissoclinamide 9 and 10, patellamides A−E and ulithiacyclamides are described as metal-binding compounds. Ascidiacyclamide was the first cyanobactin to be described as a metal binder. The characterized bis-copper (II) complex of ascidiacyclamide revealed two copper ions separated by a bridging carbonate anion fixed in the “saddle-shaped” molecule [80]. Likewise, studies regarding the binding of Cu(II) to patellamide D, which presents a “figure of eight” conformation in the solid state, determined a similar associated bis-copper complex [81]. Patellamides A−C were able to selectively bind to Cu(II) and Zn(II), but not to Ca(II) or Mg(II). These cyanobactins have the ability to bind two metals per molecule, and patellamide B and E experience a conformational change from the “figure of eight” to the “square” upon copper complexation [82]. In another study, Morris and colleagues [83] demonstrated that patellamide C presented extreme selectivity for Cu(II), even in the presence of excess Zn(II). In contrast, the same cyanobactin was not able to significantly bind to Co(II), Ni(II) and Hg(II). The higher affinity of patellamide C to Cu(II) over Zn(II) seems to be linked to the availability of the exposed nitrogen-binding sites for these ions. Ulithiacyclamide shows identical selectivity for Cu(II) and immediately forms Cu(II) complexes without any change in its conformation. Patellamide A, which mostly presents the “square” form in solution, was less selective for copper and the binding resulted in small conformational changes. Interestingly, patellamide C binding of Cu(II) led to a conformational change from the “figure of eight” to the “square,” allowing the favorable Cu(II) accommodation [83]. It seems clear that the metal-binding activity is dependent on the molecular shape. Cu(II) prefers the “square planar” or “square pyramidal” environments provided when the patellamides assume the “square” conformation. On the other hand, Zn(II) prefers a “tetrahedral” binding environment, that is not available in patellamide C. Nevertheless, the binding of Zn(II) does take place but it is probably less ideal than the binding of Cu(II) [83]. These studies revealed that copper is the biologically relevant metal for these cyanobactins. In addition to molecular shape, other factors seem to affect the interaction of cyanobactins with metals. The nature of the anions, the presence/absence of a base to deprotonate the cyclic peptide, the reaction solvent and the nature of the heterocycles that constitute the compounds are important factors that influence metal-binding complex formation [68]. Similar studies have been performed on lissoclinamides 9 and 10, revealing that lissoclinamide 10 is more selective for Cu(II) than lissoclinamide 9, even in the presence of excess Zn(II). Additionally, it was discovered that both cyanobactins present specific Cu(II)-binding motifs that are very close to the geometrical ideal [84].

It is possible that the natural role of ascidiacyclamide and patellamides includes Cu(II) coordination. However, cyanobactin metal-binding constants are usually low, when compared to physiologically relevant molecules. Furthermore, metal-binding constants were determined *in vitro* and hence determination of this in natural environments would be important.

Several biological functions have been proposed for the Cu(II) complexes of cyclic peptides. Those include: Cu(II) transport and storage, copper detoxification, CO_2_ hydration (carbonic anhydrase reactivity), oxygen activation and phosphoester hydrolysis (phosphatase reactivity). Additionally, Cu(II) cyclic peptide complexes may also act as enzyme co-factors [85].

The recent studies by Comba [85] suggest that the possible biological function of the patellamides produced by an organism is the carbonic anhydrase activity of their dicopper complexes. The dicopper(II)-patellamide complexes seem to be the first structural, spectroscopic and competent functional models for a carbonic anhydrase based on copper. However, it is important to point out that to date, there is no substantial evidence that Cu(II) complexation of patellamides is relevant for the ascidians [85].

Therefore, the essential question regarding the ecological roles of cyanobactins remains to be answered. It seems possible that cyanobactins may have several roles in nature and continuous efforts will be necessary to provide new insights concerning this intriguing subject.

## 9. Biotechnological Importance

Cyclic peptides offer several advantages over linear peptides for their pharmaceutical potential due to versatility, higher permeability across membrane barriers, greater resistance to enzymatic degradation, enhanced bioavailability [86,87] and higher affinity in receptor binding [87]. Mid-sized macrocycles and cyclic peptides can interact with extended binding sites that are not acquiescent to small molecules and have several conveniences compared to the native proteins and antibodies [1]. The biotechnological capability of cyanobactins’ biosynthetic pathways relies essentially on the guided macrocyclases and precursor peptide biosynthesis, and mobility of recognition sequences (RSs) [33,36,40,88]. Cyanobactins are directly encoded in the precursor peptide sequence(s), which are being defined by a set of adaptable tailoring enzymes. These proprieties make them very promising for bioengineering the compounds of interest, since the precursor peptide sequence(s) can be easily manipulated in order to produce the recombinant compounds.

The transportable nature of the leader peptide has already been demonstrated through the heterologous production of cyanobactins. For example, patellamides were heterologously produced by a microcin-like pathway in *P. didemni* [8] and by shotgun cloning [7]. Modification of the precursor peptide variable sequence was successfully achieved leading to the production of eptidemnamide in *E. coli*, a new cyclic peptide similar to an anticoagulant used clinically [34]. Likewise, the heptapeptide trunkamide was also produced in *E. coli* by co-expressing the mechanisms involved in the production of the hexapeptide patellin 2 and the octapeptide patellin 3, with the precursor peptide for trunkamide [5]. The cyanobactin biosynthetic pathway *tru* was engineered in order to integrate multiple tandem mutation and non-proteinogenic amino acids, using eight heterologous components simultaneously expressed in *E. coli*. Ptru-SD1 vector, encoding for the *tru* biosynthetic pathway, was cloned in *E. coli* together with vector ptruE, encoding for new cyanobactins. As a result, 16 out of the 22 recombinant compounds were expressed. In addition, patellamide precursor peptides responsible for ulithiacyclamide and patellamide C synthesis were cloned into the *tru* pathway in order to combine *tru* and *pat* biosynthetic pathways. As a result, one of the derivatives containing two thiazoline residues was successfully synthesized, thus presenting the first report of a *tru* derivate containing this modification [88]. Later, in order to determine the acceptable mutations in the trunkamide pathway, several random double and quadruple mutants were synthesized and expressed in *E. coli*. More than 300 new compounds were obtained as a result of the successful incorporation of 323 amino acids in 159 sequences, demonstrating the potential of the *tru* pathway in drug discovery, synthetic biology and biotechnology [89]. Conclusively, the use of the leader peptide and its ability to tolerate a broad range of biosynthetic enzymes allows the production of engineered compounds in heterologous hosts, thereby emphasizing its potential [3].

The cyanobactin pathways, *pat* and *tru*, appear to be unbiased by mutations. Enzymes are directed by short recognition sequences enclosed by substrates that can be substituted with the intention of effectively guiding the inclusion of specific functional groups to the mature cyanobactin. In order to test the mobility of post-translational modifications, hybrid precursor peptides comprising sections of *tru*, *lyn* and *pag* genetic clusters were constructed, leading to the production of the natural products in *E. coli*. The swapping of recognition sequences raises an interesting approach for synthetic biology concerning the tailoring of peptide products *in vitro* and *in vivo* [36].

A number of studies have been focusing on PatG activity with the aim to synthesize cyclic peptides [33,40,90]. Small libraries were also synthesized *in vitro* using distinct substrates for the prenyltransferase enzymes [39]. The discovery of the PatG macrocyclase domain [40], as well as the PatG macrocyclase crystal structure [33], allowed for the elucidation of the key features involved in substrate recognition and reactivities of this enzyme. Nevertheless, one of the major limitations of PatG is its slow enzyme activity. In that sense, studies exploring the PatG biotechnological potential started to arise and have focused on attempts to increase the catalytic efficiency of this enzyme. The engineering of the PatG macrocyclase domain from the patellamide pathway has enabled the macrocyclization of two peptides containing a mutated macrocyclase signature (AYRG) that are not synthesized by the wild-type enzyme. The reaction rate of this enzyme was also an order of magnitude faster than that in the wild-type PatG macrocyclase with a non-mutated macrocyclase signature (AYDG) [40]. In another study, an engineered PatG variant with mutations in the macrocyclase domain, named PatG^subtiligase^, was generated. In the presence of Ala-Gly, the PatG^subtiligase^ was more efficient in catalyzing the cyclization via aminolysis than the wild-type enzyme. This reaction resulted in the production of the cyclic peptides without adducts. In contrast, the wild-type enzyme reaction displayed linear and cyclic peptides as well as linear adducts [33].

Recent progress in the chemistry of heterocyclization using PatD and homologs [36,43,91] has allowed the synthesis of short heterocycle-containing peptides, providing a simple method to generate native-like substrates for subsequent enzymes. An *in vitro* biosynthesis system for azoline-containing peptides, designated as FIT-PAD (flexible *in vitro* translation), was conceived through the integration of a cell-free translation system (composed by RNA polymerase, translation factors and ribosome) with the cyclodehydratase, PatD. In addition, extensive mutagenesis and deletion analysis were performed on PatE. This system interestingly enabled a more efficient synthesis of an extensive variety of azoline-containing peptides expressed from synthetic DNA templates. In addition, the substrate recognition determinants for PatD catalysis were identified, disclosing the biotechnological potential of the FIT-PAD system [91]. Recently, engineering studies on LynD, from the aesturamide pathway allowed the construction of a variant of this enzyme that efficiently processes substrates without a leader peptide, representing a great potential in biotechnology. The engineered LynD can act on peptides without the leader sequence and only three *C*-terminal residues (AYD) are necessary for those products where macrocyclization is required [92].

As previously mentioned, a more desirable approach for the synthesis of cyclic peptides is the successful introduction of tailored reactions that can significantly modify the potentially pharmaceutical peptides. The synthesis of azol(in)e-based cyclic peptides has already been reported. In this study, simple derivatives of the patellamide core sequence were synthesized *in vitro* through a new universal method [48]. More recently, cyanobactin-tailoring enzymes have been investigated *in vitro* in order to produce new natural compounds with tailored post-translational modifications. This was achieved using hybrid biosynthetic structures encompassing a mixture of enzymes, precursors and RS elements from trunkamide, prenylagaramide, cyanothecamide, patellamide, and aesturamide pathways. The synthesis of highly unnatural derivatives such as the N-C peptide macrocycle (22 amino acids in length) was accomplished by manipulating the order of addition and individuality of the enzymes. This study also demonstrates for the first time the reconstitution of a native, multistep RiPP pathway with multiple enzymes in one pot [93].

The continuous discovery of new genetic clusters [11] as well as the promiscuity of the tailoring enzymes involved in cyanobactin production suggest that investigations of their biotechnological potential should be continued in order to meet the pharmaceutical requirements and further contribution to the development of new compounds.

## 10. Conclusions

Cyanobactins constitute one of the largest classes of metabolites, with more than 100 cyanobactins known from free-living as well as symbiotic cyanobacteria, including symbionts of sponges and ascidians. Genome-mining techniques empower the continuous finding of these small peptides, and reveal that uncovered cyanobactins warrant further investigation. Several genetic clusters and their associated compounds have been described from a diverse range of cyanobacteria species, but the bioactivities or ecological roles of most of these compounds are still unknown. Since the first report of the patellamide pathway, *pat*, cyanobactin gene clusters have revealed an unexpected arrangement, especially in terms of the presence of several precursor peptides, encoding for different compounds, as well as the absence of heterocyclase and oxidase enzymes. Cyanobactins are known for their attractive bioactivities as disclosed thus far. Still, several issues remain concerning their relevant use in the development of new clinical drugs. Their mechanism of action needs to be understood in order to prioritize their application for the appropriate target. Moreover, the amount of cyanobactin obtained from cyanobacterial cultures is strain dependent and, for this reason, efforts in biotechnological approaches need to be pursued, particularly in terms of the promiscuity of tailoring enzymes. Furthermore, unraveling the ecological roles of cyanobactins, especially metal-binding ones, will be important for biotechnology development, since it may enable the design of synthetic analogs with a high affinity for reagents.
